# Decoding the Plant Growth Promotion and Antagonistic Potential of Bacterial Endophytes From *Ocimum sanctum* Linn. Against Root Rot Pathogen *Fusarium oxysporum* in *Pisum sativum*

**DOI:** 10.3389/fpls.2022.813686

**Published:** 2022-02-14

**Authors:** Shikha Gupta, Sangeeta Pandey, Satyawati Sharma

**Affiliations:** ^1^Amity Institute of Biotechnology, Amity University Uttar Pradesh, Noida, India; ^2^Amity Institute of Organic Agriculture, Amity University Uttar Pradesh, Noida, India; ^3^Centre for Rural Development and Technology, Indian Institute of Technology Delhi, New Delhi, India

**Keywords:** endophytes, plant growth promotion, biological control, volatile compounds, induced systemic resistance, *Fusarium* root rot

## Abstract

The present study demonstrates plant growth promotion and induction of systemic resistance in pea (*Pisum sativum*) plant against *Fusarium oxysporum* f.sp. *pisi* by two bacterial endophytes, *Pseudomonas aeruginosa* OS_12 and *Aneurinibacillus aneurinilyticus* OS_25 isolated from leaves of *Ocimum sanctum* Linn. The endophytes were evaluated for their antagonistic potential against three phytopathogens *Rhizoctonia solani*, *F. oxysporum* f. sp. *pisi*, and *Pythium aphanidermatum* by dual culture assay. Maximum inhibition of *F. oxysporum* f. sp. *pisi* was observed by strains OS_12 and OS_25 among all root rot pathogens. Scanning electron microscopy of dual culture indicated hyphal distortion and destruction in the case of *F. oxysporum* f. sp. *pisi*. Further, volatile organic compounds (VOCs) were identified by gas chromatography–mass spectrometry (GC-MS). The GC-MS detected eight bioactive compounds from hexane extracts for instance, Dodecanoic acid, Tetra decanoic acid, L-ascorbic acid, *Trans*-13-Octadecanoic acid, Octadecanoic acid. Both the endophytes exhibited multifarious plant growth promoting traits such as indole acetic production (30–33 μg IAA ml^–1^), phosphate solubilization, and siderophore and ammonia production. Pot trials were conducted to assess the efficacy of endophytes in field conditions. A significant reduction in disease mortality rate and enhancement of growth parameters was observed in pea plants treated with consortium of endophytes OS_12 and OS_25 challenged with *F. oxysporum* f.sp. *pisi* infection. The endophytic strains elicited induced systemic resistance (ISR) in pathogen challenged pea plants by enhancing activities of Phenylalanine ammonia lyase (PAL), peroxidase (PO), polyphenol oxidase (PPO), ascorbate oxidase (AO), catalase (CAT) and total phenolic content. The endophytes reduced the oxidative stress as revealed by decrease in malondialdehyde (MDA) content and subsequently, lipid peroxidation in host plant leaves. Robust root colonization of pea seedlings by endophytes was observed by scanning electron microscopy (SEM) and fluorescence microscopy. Thus, plant growth promoting endophytic *P. aeruginosa* and *A. aneurinilyticus* can be further exploited through bio-formulations for sustainable protection of crops against root rot diseases as bio-control agents.

## Introduction

The conventional agricultural practices heavily rely on bulk use of chemical fertilizers and pesticides resulting in environmental pollution and deterioration of soil and human health. Plant- microbe interactions are one of the most widely studied phenomena offering huge possibilities to design a tailormade formulations of microbes as a substitute to chemical inputs in enhancing plant growth and development.

In this regard, the use of plant-associated microbiota, collectively known as endophytes, represents a reliable and sustainable farming approach for plant growth as well as phytopathogen growth suppression and disease control. Endophytes are those microorganisms which invariably colonize internal plant tissues without causing any apparent disease symptoms in host plants (Hardoim et al., 2015). There are numerous studies demonstrating the immense potential of endophytes in plant growth promotion, stimulation of stress tolerance, suppression of plant pathogenic diseases, and alleviation of negative effects manifested upon biotic and abiotic stressed conditions through various direct and indirect mechanisms (Etminani and Harighi, 2018; Aeron et al., 2020; Kushwaha et al., 2020).

These mechanisms include nitrogen fixation, plant growth hormone (auxins, cytokinin, and gibberellins) production, solubilization of phosphates and sequestration of iron by production of siderophores, production of antimicrobial bioactive metabolites, and competition for nutrients and ecological niches (Latha et al., 2019).

Endophytic bacteria endowed with another prominent role, i.e., elicitation of induced systemic resistance in plants whereby the plant’s innate defensive system is reprogrammed in a positive manner to counterattack pathogen challenges (Jacob et al., 2020). The plant defensive system is linked with the elicitation of phenylpropanoid pathway that may result in enhancing the activities of various defense-related enzymes such as Phenylalanine ammonia lyase (PAL), peroxidase (PO), polyphenol oxidase (PPO), ascorbate oxidase (AO), inducing antioxidants like catalase (CAT), and stimulating the accumulation of phenolic compounds and thus, manage pathogenicity and disease caused by pathogens in plants (Kloepper and Ryu, 2006). The promising role of plant growth-promoting bacterial endophytes in augmenting induced host resistance for controlling further pathogen attack was documented in previous reports (Rashid et al., 2017; Nandhini et al., 2020).

*Fusarium oxysporum*, a species complex with different *Formae speciales* is a soil-borne phytopathogen responsible for vascular wilt, root, and crown rot diseases on a diverse array of economically important crop plants such as tomato, banana, sweet potato, cotton, pea, chickpea (Gordon, 2017; Edel-Hermann and Lecomte, 2019). *F. oxysporum* Schl. f. sp. *pisi* Snyd. and Hans. causing vascular wilt and root rot diseases are a major threat that hampers agricultural productivity resulting in enormous yield and economic losses in infected pea crops (Porter et al., 2015; Bani et al., 2018). Endophytic biocontrol agents with multifarious PGPR characteristics have been successfully isolated from different plant parts such as *Bacillus amyloliquefaciens* FBZ24 (Elanchezhiyan et al., 2018), *Burkholderia gladioli* E39CS3 (Ahmad et al., 2021), *Burkholderia cenocepacia* 869T2 (Ho et al., 2015), *Enterobacter cloacae* SM10 (Tsuda et al., 2001) *Bacillus tequilensis* (Bhattacharya et al., 2019), *Bacillus subtilis* (Baysal et al., 2008), *Bacillus velezensis* B-36 (Wang et al., 2020), *Pseudomonas lini* (Muñoz Torres et al., 2021), and proactively used to ward-off the *Fusarium* wilt and root rot infection in different crops.

In this context, the present study was designed to explore the antifungal activity of bacterial endophytes from ethnomedicinal plant *O. sanctum* Linn. against *F. oxysporum* f sp. *pisi* underlining the beneficial therapeutic values of host plants in traditional Unani and Ayurveda medicine. The possible mechanism of endophytic bacterial strains involved in the *in vitro* antagonism against *F. oxysporum* f sp. *pisi* in dual cultures were determined such as phenotypic exhibition of multiple plant growth-promoting and biocontrol traits, production of antifungal volatile organic compounds. Secondly, *in planta* assay on pea plants was carried out to examine the effect of tripartite interaction (bacterial isolate-host plant–pathogen) on pea growth and development. Finally, the morphological, physiological, and biochemical parameters of pea plants grown under pathogen *F. oxysporum* f sp. *pisi* stressed soil were assessed to explore mechanistic insights on the mode of defensive action of selected endophytic bacterial isolate.

## Materials and Methods

### Sampling Site and Isolation of Endophytic Bacteria

Five plants of *Ocimum sanctum* were collected from the Sanjay Van forest area located in South-Central Ridge (28°32′00′′N 77°10′40′′E) in the National Capital Territory of Delhi in India (Supplementary Figure 1). Healthy leaves without any visible damage were detached and safely brought to the laboratory in zip lock bags. The leaf segments were thoroughly washed in running tap water to remove adhering soil particles prior to surface sterilization process as per Zinniel et al. (2002). In brief, the leaf tissues were washed in 70% (v/v) ethanol for 1 min followed by immersion in 1% (v/v) sodium hypochlorite for 1 min and washed five to six times with sterile double autoclaved distilled water to remove disinfectant agents. Surface sterilization was confirmed by plating the aliquots of water from the final rinse onto the nutrient agar media plate which served as a control and was observed for growth after the incubation at 28°C for 48 h (Liaqat and Eltem, 2016). The surface sterilized plant tissues were macerated in phosphate saline buffer using sterile mortar pestle and serial dilution up to 10^–5^ and 10^–6^ were made. Aliquots of appropriate dilutions were plated on nutrient agar media and incubated at 28°C for 48 h. The morphologically distinct individual colonies were selected and purified by sub-culturing for further use.

### Source and Growth Maintenance of Fungal Plant Pathogens

Three fungal phytopathogens *viz. Rhizoctonia solani*, *F. oxysporum* f. sp. *pisi* and *Pythium aphanidermatum* were obtained from Indian type culture collection (ITCC), Division of Plant Pathology, ICAR-IARI, New Delhi, India. The pathogens were maintained by subculturing them on potato dextrose agar (PDA) slants and in potato dextrose broth (PDB) for further investigations.

### *In vitro* Screening of Isolates for Bio-Efficacy Assays

The bacterial isolates were screened for antagonistic activity against fungal phytopathogens through *in vitro* dual culture assay. Briefly, a loopful of the overnight grown bacterial culture was streaked symmetrically around the 5-mm diameter agar plug of fungal mycelium placed in the center of potato dextrose agar (PDA) medium. The plates were incubated for 7 days at 26°C and examined for inhibition zones against fungal pathogens. Plates with only a mycelial plug of pathogen at the center of the PDA plate served as controls. All experiments were conducted in triplicates.

### Scanning Electron Microscopy Analysis of Antagonistic Effect of Bacterial Endophytes on *Fusarium oxysporum*

The mycelial plugs of *F. oxysporum* f. sp. *pisi*, incubated in bacterial suspension for 2 days at 200 rpm, were taken as specimens for observations under SEM. The fungal mycelium was placed on the glass cover slips and fixed in 1.5% glutaraldehyde in 0.05 M phosphate buffer (pH 7.3) for 4 h at 4°C. Followed by fixation, the specimen was washed three times with phosphate buffer for 10 min., post fixation of the samples was carried out using 1.0% OsO_4_ in 0.05 M phosphate buffer (pH 7.2) at 4°C for 4 h and then subsequently washed three times with distilled water. Then, the samples were dehydrated with increasing concentration of ethanol from 30 to 100% ethanol (v/v) at 10 min interval. A similar procedure was done with fungal mycelium taken from the petri plate grown without bacterial inoculation (control). Followed by dehydration, the dehydrated samples were critical-point dried, mounted on SEM stubs using carbon tapes, and coated with gold: palladium (60:40) for visualization under ZEISS EVO scanning electron microscope and photomicrographs were recorded (Jang et al., 2011).

### Antifungal Assay of Volatile Organic Compounds

The *in vitro* production of inhibitory volatile compounds by selected endophytes against *F. oxysporum* f. sp. *pisi* was qualitatively assessed as per Yuan et al. (2012). Briefly, 0.1 mL of the selected bacterial broth culture was plated on nutrient agar medium while the mycelium of *F. oxysporum* f. sp. *pisi* (7 mm diameter) from actively growing culture was cut and placed on the center of the fresh PDA plate. The partitioned plates containing only fungal mycelium, but no bacterium, was served as control. The PDA plate containing mycelial plug was kept inverted over the nutrient agar plate with bacterial culture and sealed firmly with parafilm. The plates were incubated at 28°C for 7 days and inhibition of fungal growth were measured.

### Gas Chromatography–Mass Spectrometry Analysis of Volatile Compounds in Bacterial Extract

The methanolic bacterial extracts were prepared with overnight grown culture of the respective bacterial strains, centrifuged at 10,000 rpm for 5 min and the resulting precipitate was dissolved in HPLC grade methanol. The methanolic extract was then subjected to vacuum evaporation on a rotatory evaporator. The resulting residue was suspended in 10 mL methanol and then fractionated with equal amounts of n-hexane. The hexane faction was evaporated and the residue ∼1 g was re-dissolved in 10 mL *n*-hexane. The final extracted hexane solution was filtered through 0.2 μm sterilizing-grade filters into HPLC vial and analyzed through Thermo scientific TSQ 9000 gas chromatograph interfaced with a triple quadrupole mass spectrometry to detect volatile biomolecules. The volatile compounds were identified using National Institute of Standards and Technology (NIST) library of mass spectra (Slama et al., 2019).

### Molecular Characterization of Bacterial Endophytes

The genomic DNA from the selected antagonistic bacterial endophytes was obtained according to the conventional phenol/chloroform method described by Sambrook et al., 1989. The 16S rRNA gene was amplified using two universal primers 16SF (5′-AGA GTT TGA TCC TGG CTC AG-3′) and 16SR (5′-AAG GAG GTG ATC CAC CGC A-3′) by polymerase chain reaction (PCR) with Bio-Rad thermo cycler (Bio-Rad, Hercules, CA, United States) using an initial denaturation step at 94°C for 5 min followed by 35 cycles of denaturation at 94°C for 1 min, annealing at 48°C for 1 min, extension at 72°C for 1 min and final polymerization step at 72°C for 10 min. The amplified DNA fragments were sequenced by Sanger dideoxy sequencing method and resultant sequence was subjected to web-based identification tool on EzBioCloud database^1^. The phylogenetic tree was developed by Neighbor- joining method using MEGA program, version 10.0.

### *In vitro* Studies of Bacterial Endophytes for Plant Growth Promoting Traits

The production of IAA of isolates was analyzed by mixing cell free supernatant with Salkowski’s reagent (35% perchloric acid + 0.5M FeCl3) and quantified spectrophotometrically at 530 nm with the help of standard curve of pure Indole acetic acid (IAA, Hi-MEDIA) (Ahmad et al., 2008). The phosphate solubilizing activity was qualitatively evaluated using Pikovaskya’s agar plates supplemented with 2% (w/v) insoluble inorganic Tricalcium phosphate [Ca_3_(PO_4_)_2_, Hi Media] (Nautiyal, 1999). Furthermore, the solubilized phosphate (Soluble P mg/L) was quantified in NBRIP medium using standard curve of KH_2_PO_4_ (HI MEDIA) as per Fiske and Subbarow (1925). The potential ability of ammonia production was estimated by using Nesslar’s reagent in accordance with Ahmad et al. (2008). Non-inoculated medium was served as control. For HCN production, the selected bacterial isolates were streaked on nutrient agar medium supplemented with 0.4% glycine. A Whatman filter paper soaked in picrate solution (2% Na_2_CO_3_ + 0.5% picric acid) was placed on the upper lids of Petri plates and monitored for 4 days for the development of orange to red color which indicated cyanogenic activity of isolates (Lorck, 1948). The production of siderophores was determined on the Chrome Azurol S (CAS) agar medium as described by Schwyn and Neilands (1987). Development of orange-yellow halo around the bacterial colonies was considered as positive.

### *In planta* Assay for Plant Growth Promotion and Bio Efficacy Against *Fusarium oxysporum*

#### Planting Materials and Sterilization Process

Pea seeds were obtained from Division of vegetable science, Indian Agricultural Research Institute (IARI), Pusa, New Delhi, India. Seeds were surface sterilized in 70% (v/v) ethanol for 1 min followed by 10 min submergence in 1% (v/v) sodium hypochlorite solution (NaClO) and then washed six–seven times with deionized water.

#### Preparation of Bacterial Inoculum and Seed Bacterization

The bacterial cell suspensions were prepared by aseptically inoculating pure bacterial cultures in LB growth medium and incubated overnight at 28°C. The bacterial cells were harvested by centrifugation at 5,000 *g* for 5 min and the resultant pellet was washed with sterile distilled water (two–three times). The pellets were finally diluted with sterile 0.03 M MgSO_4_ and adjusted to concentration 10^8^ cfu ml^–1^ as measured with spectrophotometer at 600 nm. For consortia development, compatibility test between the strains was carried out on nutrient agar plates (Supplementary Figure 2). The consortium of bacteria was prepared by inoculating overnight grown bacterial cultures of OS_12 and OS_25 in fresh nutrient medium in the ratio of 1:1 and incubated for 24 h at 120 rpm at 28°C. The seed bacterization was carried out by immersing the sterilized pea seeds in bacterial culture suspension prepared in sterile 0.03 M MgSO_4_ for 30 min and then air dried in laminar air flow for 2 h.

### Effect of Bacterial Endophytes on Pea Germination

The germination test was carried out by aseptically placing 10 bacterized and uninoculated seeds on Whatman filter paper in 10 cm Petri dishes (three replicates per treatment) as per treatment conditions and incubated in the growth chamber with optimum light and temperature conditions, i.e., 16:8 light: dark photoperiod at 28°C. The paper was moistened with normal tap water (control). The Petri dishes with uninoculated seeds served as control. Following 10 days incubation, germination percentage was recorded.

### Soil Preparation and Analysis

The soil was collected from the organically cultivated agricultural field and sieved (2 mm pore size) to remove any debris and large aggregates. The soil was mixed with farmyard manure in the ratio of 4:1 (w/w) and two times autoclaved at 121°C for 30 min at 24 h intervals. The sterilized soil was stored in the Ziplock bags at 4°C in the laboratory and analyzed for its physicochemical properties in triplicates for plant growth study. The soil with sandy loam texture has electrical conductivity (0.0354 ds m^–1^), pH (4.5), organic C (0.58 g kg^–1^), N (0.19 g kg^–1^), P (0.02 g kg^–1^), K (0.23 g kg^–1^).

### Preparation of Pathogenic Inoculum

The mycelial plugs of ∼5 mm from the *F. oxysporum* f. sp. *pisi* PDA plate were inoculated into sterile 100 mL potato dextrose broth (PDB) medium and incubated for 7 days at 25°C without shaking. Following incubation, the broth medium was filtrated with sterile water to harvest the mycelial mats formed on the upper surface of the culture medium. The mycelial mats so collected was mixed and allowed to grow on the mixture of soil and coco peat (4:1 w/w/) in the proportion of one piece of mat to 4 kg soil mixture for mass multiplication for 2 weeks at 25°C.

### Experimental Design and Pot Trials

The experimental design consisted of two sets of experiments with four treatments each under normal and pathogenic challenged conditions to evaluate plant growth and biocontrol potential, respectively, of selected endophytes. The four different treatments were applied to the sterilized seeds of pea in the experiment were: (i) Control, (ii) Strain OS_12 inoculated, (iii) Strain OS_25 inoculated and (iv) Dual Consortia (OS_12 + OS_25) inoculated. Untreated seeds inoculated with pathogen, *F. oxysporum* f. sp. *pisi* served as positive control while those without fungus inoculation served as negative control. Three replications were maintained for each treatment and the experiment was repeated thrice.

The bio primed pea seeds were then sown in plastic pots (20 cm × 20 cm) filled with sterile soil mixture (2.5 kg pot^–1^) at a rate of 4 seeds per pot at a depth of 1.5–2.0 cm. In total, 36 seeds were sown for each treatment. After 4 weeks of seedling emergence, seedlings were divided into two sets, one was allowed to grow under normal conditions while the other one grew under pathogen challenged conditions to determine the potential of isolates to promote plant growth and resist biotic stress. The inoculum of phytopathogen, *F. oxysporum* f. sp. *pisi* was added in the potting mixture at the rate of 50 g per pot to produce fungal infested soil. The pots were watered daily to maintain favorable moisture level.

### Assessment of Plant Growth Parameters and Disease Severity

After 14 days, following pathogen challenge, the plants were carefully harvested from the soil, washed with tap water and the morphological parameters related to length and fresh weight of roots and shoots of pea plants were measured. The plants were rated for disease severity on a scale from 1 to 5 based on visual damage to roots system, where 1- healthy or no symptoms to root tissue; 2- 25% damage to root tissues 3- 50% damage to root tissues, 4- 75% damage to root tissues and 5- complete damage to tissues (Bahroun et al., 2018).

### Photosynthetic Pigments Estimation: Total Chlorophyll and Carotenoids Content

The photosynthetic pigments, chlorophyll and carotenoids content of pea leaves were determined by ethanol and acetone extraction methods, respectively, as per standard procedure. The absorbance of the collected supernatant was recorded at 663 and 645 nm for chlorophyll estimation and at 460 and 510 nm for carotenoid analysis. Total chlorophyll content and carotenoid content was calculated as mg g^–1^ of Fresh weight (FW) using the following equations (Arnon, 1949; Sarker and Oba, 2018).

### Analysis of Induced Systemic Resistance in Plants

The leaf tissue sample (0.5 g) was macerated in 4 mL 0.2 M borate buffer (pH 8.7) along with 1.4 mM β-mercaptoethanol. The subsequent crude enzyme extract was taken and mixed with 500 μl borate buffer, 1 mL of 0.1 M L-phenylalanine and distilled water and incubated at 30°C for 30 min. The reaction was stopped by adding 500 μl 1 M trichloroacetic acid. The PAL activity was calculated as Units per gram of fresh weight where 1 unit of PAL activity is defined as the 0.01 change in absorbance in unit time at 290 nm (Havir, 1987).

The activity of Peroxidase was measured in leaves extract (100 μl) of all the studied treatments by adding 1.5 mL of 0.05 M pyrogallol and 0.5 ml of 1% H_2_O_2_. The increase in absorbance of reaction mixture was recorded due to oxidation of pyrogallol to purpurogallin at 420 nm at regular intervals of 20 s for 3 min and expressed as change in absorbance under the assay conditions per min per gram of fresh weight (Hammerschmidt et al., 1982).

The polyphenol oxidase (PPO) activity of leaves enzyme extract was measured at 495 nm at 30 s regular intervals for 3 min using 0.01 M catechol as a substrate and expressed as change in absorbance of 0.01 under the assay conditions per min per gram of fresh weight.

Ascorbate oxidase (AO) enzyme activity was estimated as per protocol of Drumm et al. (1972). The amount of ascorbate oxidase was measured as change in absorbance of reaction mixture (3 mL 10 mM ascorbic acid + 100 μl enzyme extract) at 265 nm and expressed as units of μmole ascorbate degraded min^–1^ mg^–1^ protein.

Catalase enzyme activity of leaves extract of all the studied treatments was estimated in terms of degradation of H_2_O_2_ at 240 nm as per protocol of Beers and Sizer (1952). The reaction mixture consisted of 50 mm potassium phosphate buffer (pH 7.0), 20 mm H_2_O_2_ and 100 μl crude enzyme extract. The amount of catalase was calculated by using molar extinction coefficient of H_2_O_2_ (36 M^–1^ cm^–1^) and expressed as μmole H_2_O_2_ min^–1^ g^–1^ fresh weight.

### Measurement of Leaf Phenolic Content and Lipid Peroxidation

The oxidative degradation of lipid biomolecules in pea plants exposed to biotic stress was quantified using TBARS (Thiobarbituric acid reactive substance) assay as per Hodges et al. (1999) and calculated in terms of MDA (Malondialdehyde) equivalents as nmol MDA g^–1^ fresh weight. The phenolic content in pathogen challenged and non-stressed pea plants was estimated quantitatively using Folin–Ciocalteu reagent and calculated as mg gallic acid equivalent (mg GAE g^–1^ fresh weight) by means of gallic acid standard calibration curve (Siddiqui et al., 2017).

### Microscopic Visualization of Endophytic Bacteria

The pea seeds were surface sterilized and subsequently bacterized with consortium of bacterial strains OS_12 and OS_25 as described previously. The inoculated and non-inoculated (control) seeds were sown in plastic cups filled with sterile soil mixture at a rate of 2 seeds per cup. After 3-weeks, roots samples were carefully harvested from control and inoculated plants.

#### Scanning Electron Microscopy

The collected tissue samples were washed twice under running water and trimmed into tiny pieces (∼0.1–0.5 cm) with a sterile razor blade. The root segments were fixed in 2.5% (v/v) glutaraldehyde and 4% (v/v) paraformaldehyde in 0.1 M sodium cacodylate buffer solution (pH 7.2) for 2 h at room temperature. Followed by fixation, tissue samples were washed thrice with phosphate buffered saline solution. The tissue segments were post fixated with 2% (w/v) osmium tetroxide (OSO_4_) solution in phosphate buffer overnight at 4°C and dehydrated in ethanol solutions of increasing concentration at 10 min intervals followed by drying in desiccators. The samples were then mounted on to SEM stubs using double coated carbon conductive tape followed by coating with gold: palladium (60:40). Photomicrographs were recorded with ZEISS EVO Scanning Electron Microscope.

#### Fluorescence Microscopy

Fine root segments (∼2–3 mm) were prepared aseptically with a sterile razor blade and fixed with formaldehyde-glutaraldehyde fixative as described above. After fixation, the samples were washed with phosphate buffered saline solution (pH 7.2). The roots segments were aseptically transferred to a clean glass slide and stained with acridine orange in phosphate buffer solution for 10 min at room temperature in the dark. Following treatment with acridine orange, the samples were washed thrice with phosphate buffer saline solution and then air dried (Batool et al., 2015). The slides were then examined under 100× objective of fluorescence microscope with green filter.

### Statistical Analysis

All data of morphological growth attributes obtained from *in planta* assay on pea were analyzed by one-way ANOVA followed by Tukey’s test with different treatment conditions considered as independent variable under pathogen challenged and non-pathogenic conditions. All the statistical analyses were calculated at significance level *p* = 0.05 through IBM SPSS Statistics software. The experiments were performed in triplicates, the mean and standard deviation were calculated using Microsoft Excel 2016. The principal-component analysis (PCA) was carried out to represent morphological growth parameters and enzymatic activity correlated to induced systemic resistance as response variables in the present analysis through SPSS software.

## Results

### Isolation and *in vitro* Biocontrol Potential of Bacterial Endophytes

A total of 25 morphologically distinct bacterial endophytes were isolated from leaves of *O.* s*anctum* Linn. plants and purified by subculturing the isolates. No bacterial growth was observed in control plates which indicate the effectiveness of surface sterilization protocol and thus, the selected bacterial isolates were considered as leaves endophytes.

The *in vitro* suppression assay revealed eight bacterial endophytes with varied levell of growth suppression of mycelium of atleast one pathogenic fungus as presented in Table 1.

**TABLE 1:** Inhibition of mycelial growth of the phytopathogens by endophytes of *Ocimum sanctum* Linn.

S. no.	Isolates	Percent growth inhibition		
		*Rhizoctonia solani*	*F. oxysporum* f. sp. *pisi*	*Pythium aphanidermatum*
(1)	OS_01	20.39 ± 0.87^a^	55.37 ± 0.71^a^	18.07 ± 0.70^a^
(2)	OS_02	0	45.25 ± 0.37^b^	0
(3)	OS_05	0	29.68 ± 1.36^c^	32.00 ± 1.39^b^
(4)	OS_07	42.36 ± 0.66^b^	0	33.35 ± 1.06^b^
(5)	OS_12	64.17 ± 0.62^c^	70.24 ± 0.52^d^	61.39 ± 0.81^c^
(6)	OS_16	0	19.35 ± 1.07*^e^*	44.28 ± 0.31^d^
(7)	OS_21	12.06 ± 0.07^d^	34.69 ± 0.32^f^	0
(8)	OS_25	59.32 ± 1.59^e^	73.59 ± 1.76^g^	67.56 ± 0.78^e^

*Plants in dual plate assay under in vitro experimental conditions.*

*Data shown as percent of growth inhibition of mycelium of fungus by bacterial strains. Values represent mean values ± standard deviation (n = 3). Different minuscules indicate statistical difference (Turkey’s post test, P < 0.05) while same letters superscript are not significantly different.*

**FIGURE 1:**
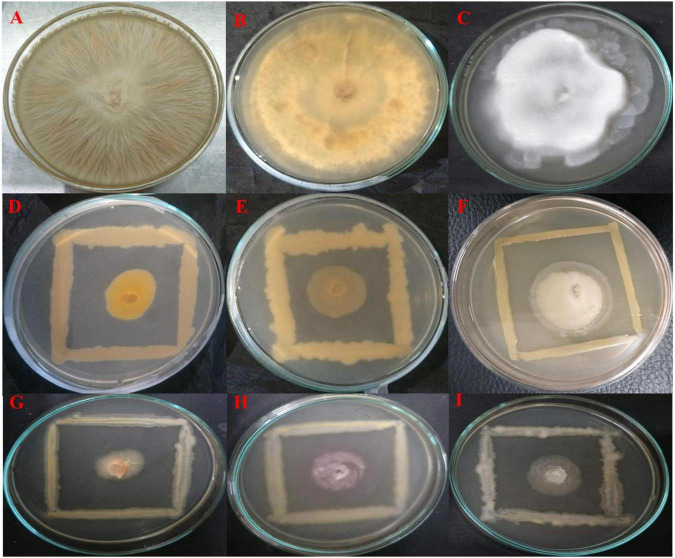
Dual culture assay for investigating *in vitro* antagonistic potential of bacterial endophytes inhibiting the mycelial growth of fungal pathogens on PDA medium after 7 days of incubation. The endophytes antagonists OS_12 **(D–F)** and OS_25 **(G–I)** have limited the growth of hyphae of pathogens with respect to control plates which showed dense hyphal growth of fungal pathogens. **(A)** *Fusarium oxysporum* f. sp. *pisi*, **(B)** *Rhizoctonia solani*, and **(C)**. *Pythium aphanidermatum*.

All endophytes indicated antagonistic potential against the mycelial growth of *F. oxysporum* except isolate OS_07. Among them, OS_12 and OS_25 significantly suppressed radial growth of *F. oxysporum* by more than 70%. The highest suppressive percent with respect to mycelial growth of *R. solani* was in the presence of isolate OS_12 (64% inhibition). Six isolates exhibited antifungal activity against *P. aphanidermatum* with isolate OS_25 displaying the highest antagonistic activity with ∼68% inhibition of hyphal growth in comparison to control. The *in vitro* antagonism assay depicted that most of the leaf endophytic isolates (∼88%) were able to limit the mycelial growth of *F. oxysporum* with mean inhibition percentage of 41% as compared to that of *R. solani* (24%) and *P. aphanidermatum* (30%). Therefore, *in planta* assay for plant growth promotion and bio-efficacy through pot trials were carried out against *F. oxysporum*. Isolates OS_12 and OS_25 were found to be most efficient antagonistic agents with respect to inhibition of mycelial growth of *F. oxysporum*, *R. solani* and *P. aphanidermatum* on PDA agar plates after 7 days of incubation (Figure 1). Therefore, isolates OS_12 and OS_25 were selected for further experimentation and characterization studies.

### Visualization of Interaction Between Antagonistic Bacterial and Fungal Plant Pathogens

**FIGURE 2:**
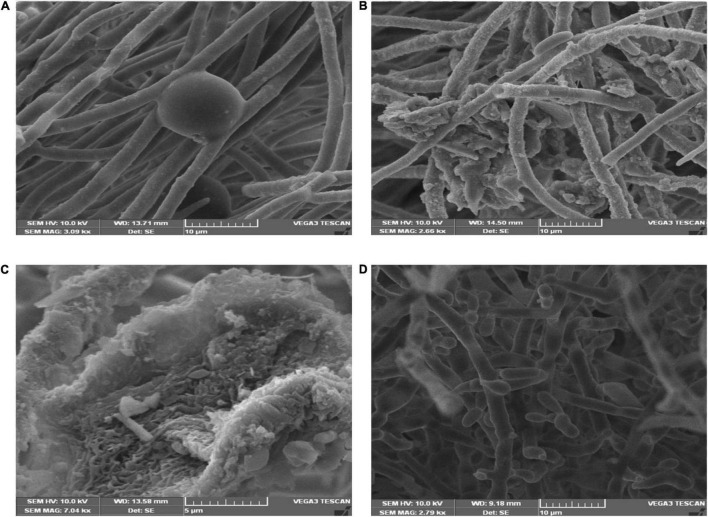
Scanning electron micrographs of mycelia of *F. oxysporum* f. sp. *pisi:* in the absence of antagonistic endophytes **(A)**. In the presence of strain OS_12 only showing dense proliferation of bacteria over the mycelial surface and hyphal destruction **(B).** In the presence of strain OS_25 only showing adhesion of bacteria to fungus mycelial structure **(C)**. SEM micrograph of consortium (OS_12 + OS_25) treated mycelia of *F. oxysporum* f. sp. *pisi* exhibiting disaggregated hyphal structures with bacterial adhesion and proliferation on mycelial surface **(D)**. Scale bar equals 10 μm **(A,B,D)** and 5 μm **(C)**.

Scanning electron microscopic studies revealed hyphal morphology of *F. oxysporum* in the presence and absence of antagonistic endophytes strain OS_12 and OS_25 (Figure 2). The control plate without antagonists indicated intact and regular structure of hyphae of *F. oxysporum* with smooth surfaces. However, micrographs clearly exhibited the irregular distortions in fungal mycelia in the presence of antagonistic strains OS_12 and OS_25 either single treated or in consortium mode.

### Antagonistic Assay of Volatile Organic Compounds and Identification by Gas Chromatography–Mass Spectrometry

**FIGURE 3:**
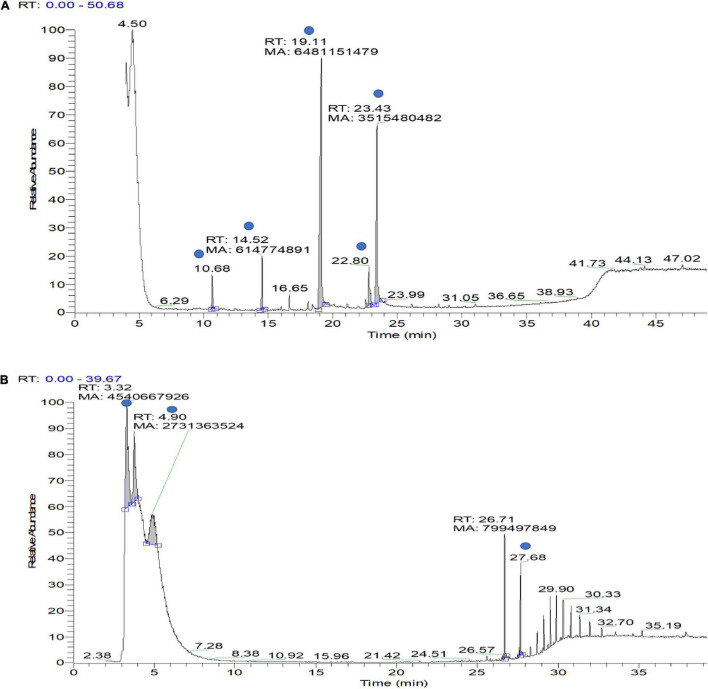
Gas chromatography–mass spectrometry (GC-MS) ion chromatogram of bioactive volatile compounds identified from hexane extract sample of bacterial isolate OS_12 **(A)** and OS_25 **(B)** with evidenced peaks (shown with blue dot) of compounds presented in Supplementary Table 1.

VOCs produced by bacterial strains OS_12 and OS_25 have significantly (*P* < 0.05) inhibited the mycelial growth of *F. oxysporum* by 35 and 24%, respectively, in comparison to control. Supplementary Table 1 has listed major peaks detected by GC-MS in the volatile fractions of bacterial isolates OS_12 and OS_25 extracted in hexane (Figure 3). For isolate OS_12, the highest percentage of peak area was observed for L-ascorbic acid with retention time 19.11, whereas the lowest percentage of the peak area (2.78) was observed for Dodecanoic acid with retention time 10.68. While in case of isolate OS_25, 2,2,3,4-Tetramethylpentane and Octadecanoic acid represented the highest (40.38) and lowest (4.49) peak area with retention time 3.30 and 27.68, respectively.

### Characterization for Multifarious Plant Growth Promoting Attributes

Indole acetic acid as confirmed by Salkowski assay, through the development of pink color of cell free supernatant. Between the two isolates, strain OS_25 showed the highest IAA production (33.8146 ± 0.38 μg ml^–1^) while the other isolate OS_12 showed IAA production of 29.3689 ± 0.26 μg ml^–1^ after the 6th day of incubation in the presence of 5 mM Tryptophan.

**FIGURE 4:**
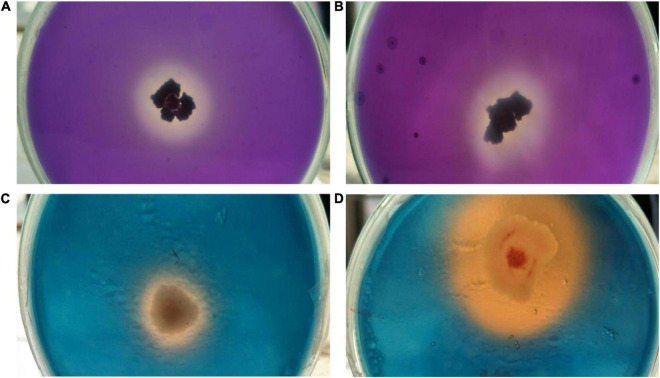
Assessment of multiple plant growth promoting traits of endophytic strains OS_12 and OS_25. **(A,B)** Zone of phosphate solubilization on NBRIP medium amended with Tricalcium phosphate. **(C,D)** Production of siderophore on CAS agar medium.

Both endophytes were able to solubilize inorganic tricalcium phosphate (2% w/v) supplemented Pikovaskya’s agar medium as visualized by clear hallow zones around the individual colonies with the highest solubilization zone of 2.8 cm by isolate OS_25 (Figures 4A,B). In quantative analysis, strain OS_25 indicated highest phosphate solubilization potential followed by strain OS_12 in NBRIP broth medium. The phosphate solubilization by strains has led to the reduction of pH of the spent medium (4.2 ± 0.02 in case of OS_25, 5.0 ± 0.01 in case of OS_12) from an initial neutral pH (7.0 ± 0.001).

Both the isolates were able to form clear orange halo zones on blue CAS agar solid medium, suggesting that strains OS_12 and OS_25 could secrete siderophores (Figures 4C,D). The selected antagonistic isolates were positive for production of ammonia as produced brown color on addition of Nessler’s reagent while failed to produce HCN.

### Molecular Characterization and Phylogenetic Analysis

**FIGURE 5:**
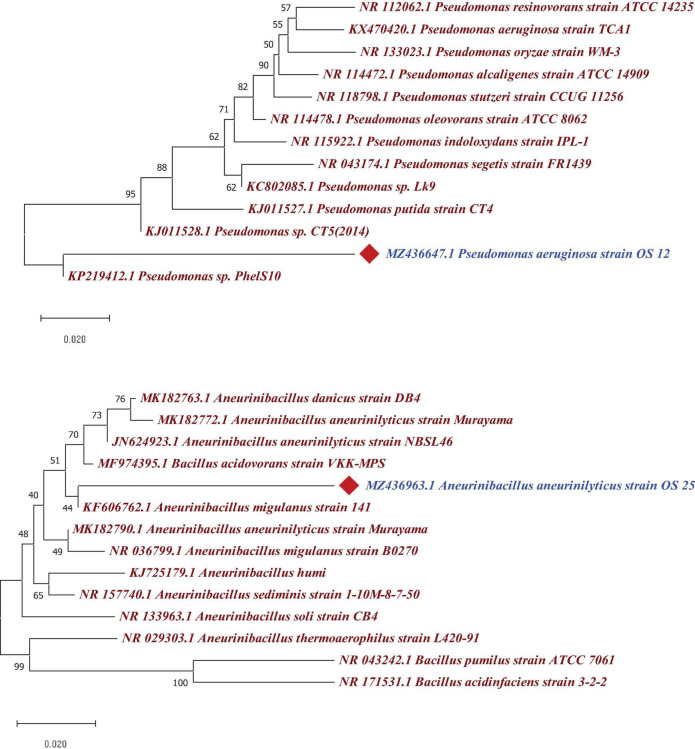
Phylogenetic tree based on a partial 16S rRNA nucleotide sequences showing the position of strains OS_12 and OS_25 with respect to other related taxa constructed with Neighbor joining method using version 10.1 of MEGA software (https://www.megasoftware.net/). The 16S rRNA gene sequences of closely related species were retrieved from NCBI GenBank databases. Evolutionary distance was computed using Maximum Composite Likelihood method. Bootstrap values (percentage of 1,000 replicates) higher than 40% are shown at node points. Bar, 0.020 substitutions per nucleotide position.

The 16S rRNA gene sequence analysis revealed that two antagonistic endophytic isolates indicated ≥90% similarity with putative strains as *Pseudomonas aeruginosa* (OS_12) and *Aneurinibacillus aneurinilyticus* (OS_25) in EzBioCloud server^2^. The 16S rRNA gene sequences of isolates OS_12 and OS_25 was submitted to NCBI GenBank database with accession numbers MZ436647 and MZ436963, respectively. The Phylogenetic analysis using MEGA X software revealed relatedness of endophytes with other strains of respective species (Figure 5).

### *In planta* Studies on Pea for Seed Germination, Plant Growth Promotion, and Bio-Efficacy

Pea seeds primed with a consortium of *Pseudomonas aeruginosa* OS_12 and *A. aneurinilyticus* OS_25 indicated maximum germination percentage in comparison to other studied treatments (Supplementary Figure 3). Further, pot trial experiments were carried out to investigate the effect of selected antagonistic bacterial bioinoculants either single or in dual combination on the growth and related attributes of pea plants after 14 days of *F. oxysporum* inoculation (Supplementary Figure 4).

### Analysis of Growth Parameters and Disease Incidence

**FIGURE 6:**
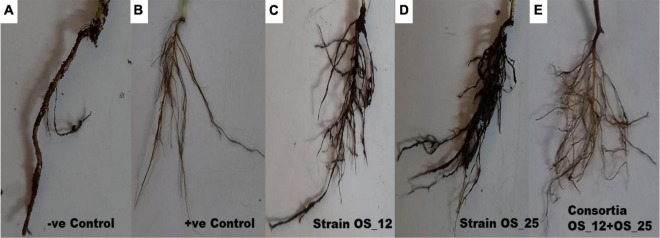
Effect of antagonistic bacterial endophytes strains OS_01 and OS_03 inoculation, single and dual combination on the root rot disease incidence after 7 days in *F. oxysporum* challenged pea plants. Severity disease symptoms in the roots system of pea plants **(A)** grown in soil infested with *F. oxysporum* only, **(B)** grown in sterile soil without infestation with *F. oxysporum*, **(C)** primed with strain OS_12 and grown in infected soil, **(D)** primed with strain OS_25 and grown in infected soil, **(E)** primed with bacterial consortia of OS_12 and OS_25 grown in infected soil.

Inoculation of bacterial endophytes in pea plants has significantly reduced the disease incidence under pathogen challenged conditions as evident from representative figures shown in Figure 6.

The highest disease incidence (∼91%) was observed in pathogen challenged control plants while lowest was recorded in plants inoculated with dual consortium. The single inoculation of OS_12 and OS_25 have also significantly reduced the symptoms of root rot disease severity by more than 70%, respectively, as compared to plants treated with *F. oxysporum* alone. However, in case of inoculated or uninoculated pea plants grown under normal (non-pathogenic) conditions, no disease symptoms were recorded (Table 2). The consortium of *P. aeruginosa* OS_12 and *A. aneurinilyticus* OS_25 was found to be capable of lowering the disease incidence as compared to other studied treatments of pea plants grown under biotic stress conditions.

**TABLE 2:** Effect of studied treatments on the incidence of root rot disease severity after 7 days of pathogen, *F. oxysporum* inoculation in pot experiments trials.

Treatments	Disease incidence percentage (%)
Positive control	0.00^a^
Negative control	91.06 ± 0.23^b^
Strain OS_12 inoculated	74.24 ± 0.59^c^
Strain OS_25 inoculated	72.35 ± 0.80^c^
Consortia (OS_12 + OS_25) inoculated	54.28 ± 0.65^d^

*Columns represent mean values ± standard deviation (n = 3). Different letters (a, b, c, d) denote statistical difference between treatments in conferring resistance to root severity disease by F. oxysporum.*

**FIGURE 7:**
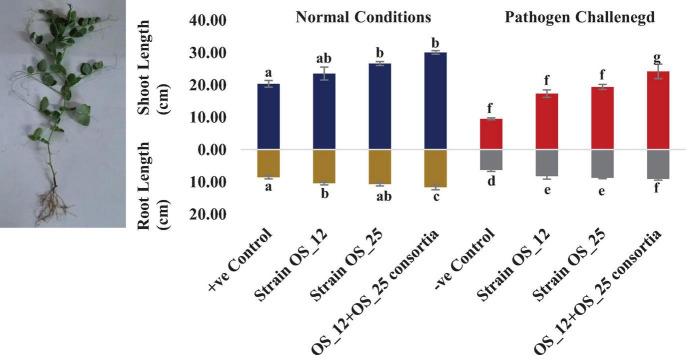
Effect of seed biopriming with endophytic biostimulant strains OS_12 and OS_25 plants on length parameter of root and shoot biomass of pea plants sown in plastic pots under normal (dark blue and dark yellow bars) and pathogenic *F. oxysporum* stress (red and gray bars) conditions. Columns represent Mean values ± standard deviation (*n* = 3 replicates per treatment). Different letters indicate statistical difference between treatments (Turkey’s posttest, *P* < 0.05) in root length and shoot length under normal and biotic stress conditions.

**FIGURE 8:**
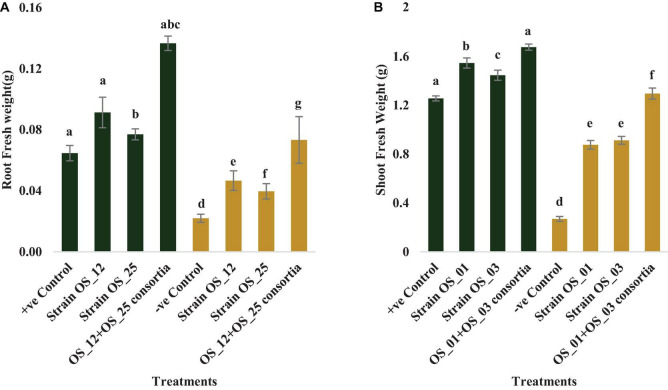
Effect of seed biopriming with endophytic biostimulant strains OS_12 and OS_25 plant on fresh weight of root **(A)** and shoot **(B)** biomass of pea plants sown in plastic pots under normal (green bar) and pathogenic *F. oxysporum* stress (purple bar) conditions. Columns represent Mean values ± standard deviation (*n* = 3 replicates per treatment). Different letters indicate statistical difference between treatments (Turkey’s posttest, *P* < 0.05) under normal and biotic stress conditions.

In comparison to control plants, OS_12 and OS_25 pre-treated plants indicated significant enhancement in root length (0.21- to 0.36-fold), shoot length (0.15- to 0.47-fold), root fresh weight (0.5- to 1.2-fold), and shoot fresh weight (0.23- to 0.34-fold) under normal conditions. *F. oxysporum* infection significantly reduced the plant growth parameters in comparison to that of non-infected plants. Overall, the plants treated with dual inoculation of endophytes OS_12 and OS_25 showed maximum increase in shoot length (1.54-fold) and root length (0.43-fold) in comparison to pathogen control plants (Figure 7). A similar trend of improvement was also observed in the case of fresh weight of root and shoot biomass of pea plants challenged with *F. oxysporum*. Among the treatments, consortium of endophytes has significantly improved the fresh biomass of roots (2.33-fold) and shoots (3.80-fold) in comparison to pathogen control plants (Figure 8).

### Analysis of Pigment: Chlorophyll and Carotenoids Content

**FIGURE 9:**
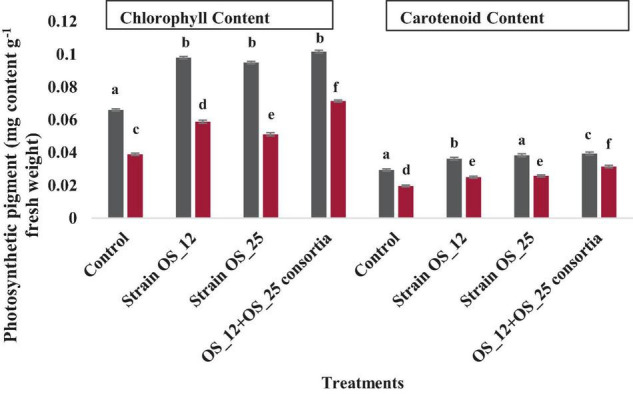
Effect of endophytic biostimulant strains OS_12 and OS_25 plant on photosynthesis associated pigments- total chlorophyl and carotenoid content of pea plants under normal (dark gray bar) and pathogenic *F. oxysporum* stress (dark red bar) conditions. Columns represent mean values ± standard deviation (*n* = 3 replicates per treatment). Different letters indicate statistical difference between treatments (Turkey’s posttest, *P* < 0.05) in enhancing chlorophyll and carotenoid content under normal and biotic stress conditions.

The infection of *F. oxysporum* caused a significant reduction of 41% in chlorophyll and 25% in carotenoid content as compared to control non-infected plants. The pea plants inoculated with individual or dual consortium of endophytes OS_12 and OS_25 has enhanced chlorophyll content in the range of 0.31–0.83- fold and that carotenoid in the range 0.27- to 0.60-fold in comparison to infected control plants. A similar enhancement in photosynthetic pigment parameters was observed in endophytes primed non-infected plants (Figure 9).

### Assessment of Defense Related Antioxidative Enzymes

**FIGURE 10:**
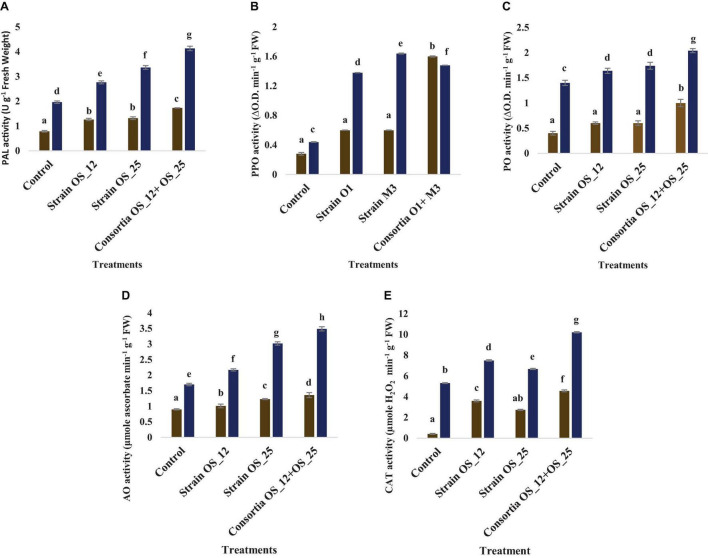
Effect of pathogen and endophytic bacterial inoculation on activities of different defense related antioxidative enzymes **(A)** phenylalanine ammonia lyase (PAL), **(B)** polyphenol oxidase (PPO), **(C)** peroxidase (PO), **(D)** ascorbate oxidase (AO), and **(E)** catalase (CAT) under different treatment conditions. Columns represent Mean values ± Standard deviation (*n* = 3). Dark yellow bar, normal conditions; blue bar, pathogen challenged conditions; Different letters indicate statistical difference between treatments (Turkey’s post test *P* < 0.05).

Inoculation with OS_12 and OS_25 has significantly increased the antioxidative (PAL, PPO, AO, and CAT) response of pea plant challenged with pathogen (Figure 10). In the presence of pathogen, phenylalanine ammonia lyase (PAL) was found highest (4.13 U g^–1^ Fresh weight) in plants inoculated with dual inoculum of OS_25 and OS_12 followed by strain (3.36 U g^–1^ Fresh weight) OS_25 and (2.76 U g^–1^ Fresh weight) OS_12 while lowest was found in uninoculated infected plants. There is no significant difference between treatments comprising single bacterial inoculation under normal conditions in comparison to control plants. Likewise, the highest level of AO enzyme (3.49 μmole ascorbate degraded min^–1^ mg^–1^ Fresh weight) and PO enzyme (2.04 change in absorbance min^–1^ g^–1^ of Fresh weight) was observed in combination treated pea plants. The polyphenol oxidase (PPO) was the significantly highest recorded by 2.72-fold in plants inoculated with strain OS_25 in comparison to pathogen control plants. The CAT activity was maximum (10.21 μmoles H_2_O_2_ min^–1^ g^–1^ fresh weight) in plants treated with consortia inoculum followed by strain OS_12 inoculated (7.5 μmoles H_2_O_2_ min^–1^ g^–1^ fresh weight) and strain OS_25 (6.68 μmoles H_2_O_2_ min^–1^ g^–1^ fresh weight). The untreated plants under pathogenic stress conditions showed the lowest enzymatic activities among all the treatments.

### Total Phenolics and Malondialdehyde Content

**FIGURE 11:**
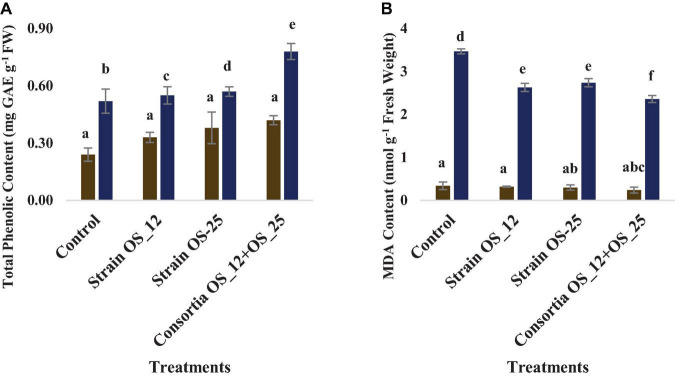
Effect of pathogen and endophytic bacterial inoculation on activities of different defense related antioxidative enzymes **(A)** total phenolic content, **(B)** malondialdehyde (MDA) content. Columns represent Mean values ± Standard deviation (*n* = 3). Blue bar, normal conditions; yellow bar, pathogen challenged conditions. Different letters indicate statistical difference between treatments (Turkey’s post test *P* < 0.05).

The total phenolics content was higher in plants treated with a combination of endophytes (0.78 mg GAE g^–1^ Fresh weight) under pathogenic stress conditions. However, no marked significant difference was observed among the treatments under normal conditions. The lowest production of malonaldehyde (MDA), measure of lipid peroxidation was observed in the plants pretreated with combination of bacterial strains (2.36 nmol g^–1^ Fresh weight) in comparison to the control plants grown without bacterial inoculation under normal (0.34 nmol g^–1^ Fresh weight) and pathogenic stress (3.47 nmol g^–1^ Fresh weight) (Figure 11).

### Microscopic Visualization of Bacterial Colonization of Pea Root

**FIGURE 12:**
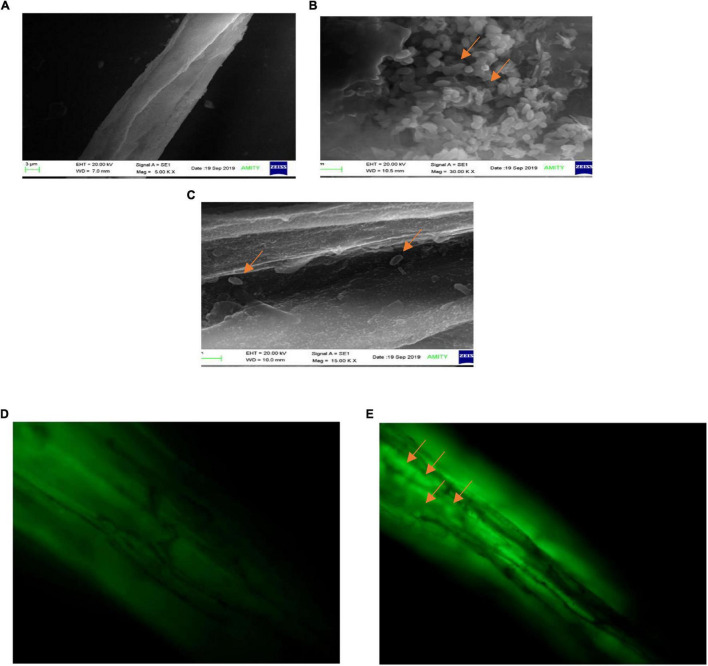
Scanning electron microscopic analysis of pea plant roots inoculated by consortia of *Pseudomonas aeruginosa* OS_12 and *Aneurinibacillus aneurinilyticus* OS_25 **(A–C)**. **(A)** Roots of non-inoculated pea plants, **(B)** bacterial cells (arrows) swarming in the vicinity of rhizoplane, **(C)** bacterial cells (arrows) in the intercellular space between epidermis and cortex of root system. Scale bar equals 3 μm **(A)**, 1 μm **(B)**, and 2 μm **(C)**. Fluorescent microscopy images of root sections stained with acridine orange **(D,E)**. **(D)** Control pea root sample without any inoculation, **(E)** root section of consortium inoculated pea plant with arrows pointing bacterial cells inside the epidermis (bar equals 10 μm).

The observations of root section from consortium treated plantlets using a scanning electron microscope revealed that the endophytic strains have colonized the roots intracellularly. The plants inoculated with consortium of *P. aeruginosa* OS_12 and *A. aneurinilyticus* OS_25 showed the presence of rod-shaped bacteria on the root surface (rhizoplane) as well as internal root tissues, and remarkably, in the intercellular spaces between the epidermal layer and the outer cortex (Figures 12B,C). Similar observations were yielded with fluorescence microscopy of root sections after acridine orange treatment which indicated small green fluorescent bacterial cells found inside the root epidermal cells (Figure 12E). The roots of uninoculated seedlings were observed undamaged with smooth epidermal root surface using scanning electron microscope (Figure 12A). Likewise, no bacteria or no auto-fluorescence was observed under green filter with root tissues of untreated control plantlets (Figure 12D).

**FIGURE 13:**
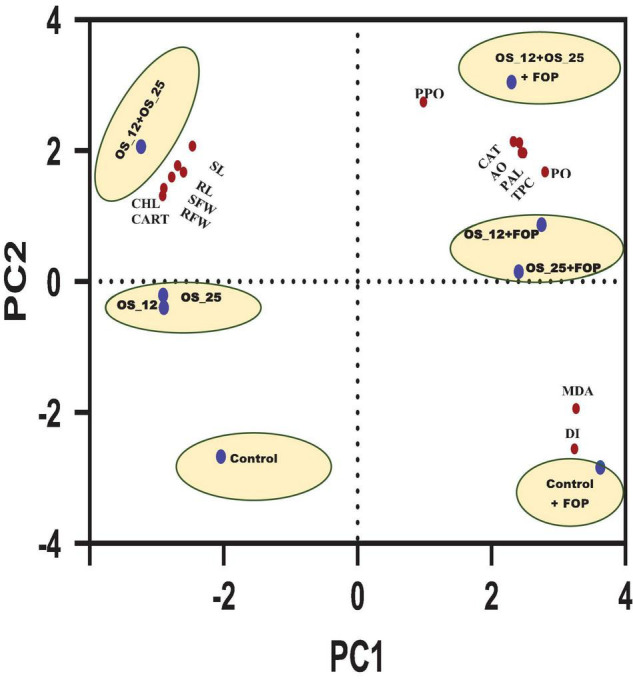
Principal component analysis of effect of different treatment (shown in circles with blue dot) with respect to control (uninoculated) and bacterial inoculation either single (OS_12 or OS_25) or in combination (OS_12 + OS_25) in the presence and absence of *F. oxysporum* f. sp. *pisi* (FOP) on growth parameters, defense enzymes and mortality rate of pea plants (shown in red dots). The *X*-axis and *Y*-axis of PCA plot represent first and second principal components (PCs). Symbols: RL, root length; SL, shoot length; SFW, shoot fresh weight; RFW, root Fresh weight; CHL, chlorophyll content; CART, carotenoid content.

Principal component analysis showed two principal components PC1 (64.49%) and PC2 (30.35%) elucidating 94.84% variance within the data set. It allowed overall distribution of treatments conditions studied in this work into six clusters as shown in PCA plot (Figure 13), with first group consist of normal control pea plants, the second with pathogen challenged control. The single bacterial treatment (*P. aeruginosa* OS_12 or *Aneurinibacillus aneurinilyticus* OS_25) formed a single cluster each under normal and *F. oxysporum* challenged conditions. A substantial difference and clustering of healthy and infected plants treated with combination of bacterial isolates was observed by PCA where morphological growth parameters, defense related enzyme activity, as well as total phenolic content were increased.

## Discussion

Endophytes are those microorganisms that spend all or at least parts of their life cycle within plant organs in mutual or some other plant-microbe relationship without causing any visible symptomatic effect on the host plant. They have been widely reported as potential antagonists against plant pathogens. Their ability to colonize internal tissues of plant and promote their growth and suppress the diseases have made endophytes an emerging tool in crop growth promotion and protection (Kandel et al., 2017).

In the present study two bacterial endophytes *P. aeruginosa* OS_12 and *A. aneurinilyticus* OS_25 from *O. sanctum* Linn. were found to suppress the growth of *F. oxysporum*. This finding was in co-relation with various published literature which reported that endophytes are involved in controlling plant pathogens (Alenezi et al., 2016; Balan et al., 2017; Kurata et al., 2017; Biessy et al., 2021; Kipgen et al., 2021).

We observed the clear distortion and abnormality in hyphal structure of *F. oxysporum* in the presence of *P. aeruginosa* OS_12 and *A. aneurinilyticus* OS_25 with scanning electron microscopy. Furthermore, as reported by Agarwal et al. (2017), Bazie et al. (2019) and Xu et al. (2020), these outcomes suggest that antibiosis tends to be the biocontrol mechanism, employed by certain species of *Pseudomonas* and *Bacillus*. In this study, the antibiosis activity has been linked to the production of antifungal volatile organic compounds, siderophore and ammonia by *P. aeruginosa* OS_12 and *A. aneurinilyticus* OS_25.

The GC-MS profiling showed eight structurally distinct compounds with some of them having previously documented plant growth promoting antimicrobial/antifungal and stress tolerance activities. Dodecanoic acid, commonly called lauric acid, is a saturated fatty acid reported to have antagonistic activity against plant fungal pathogens, *Pythium ultimum* and *Rhizoctonia solani* (Walters et al., 2003), *Aspergillus niger* (Řiháková et al., 2001), and *Fusarium* spp. (Al-Rashdi et al., 2020). Tetra decanoic acid, another saturated fatty acid commonly known as myristic acid was previously described for inhibitory activity on mycelial growth of *Alternaria solani*, *F. oxysporum* f. sp. *Lycopersici* as well as for imparting osmotic stress tolerance in plants grown in saline conditions (de Jonge et al., 2000; Liu et al., 2008). L-ascorbic acid (Vitamin C) water soluble antioxidant molecule has been reported to exhibit antifungal activity and modulate the plant defense system against pathogenic stress (Boubakri, 2017). *Trans*-13-Octadecanoic acid was also found to have pharmacological activity and anti-inflammatory activity against fungal and bacterial pathogens (Gupta and Kumar, 2017; Hussein et al., 2017). Stearic acid (Octadecanoic acid) reported to confer antagonism and enhance plant defense against microbial pathogens (Blechert et al., 1995; Henry et al., 2002; Shah et al., 2021).

Furthermore, we found out that both *P. aeruginosa* OS_12 and *A. aneurinilyticus* OS_25 possessed the ability for siderophore and ammonia production which implies their role in pathogen growth suppression and nutrient acquisition in plants (Bashan et al., 1980; Sumei et al., 2017; Bhattacharyya et al., 2020).

The bacterization of pea seeds with selected endophytic bacterial strains and consortia indicated better seedling emergence rate than the control. However, application of consortia gave better results than individual bacterial inoculation. These findings are in agreement with previously reported literature (Mishra et al., 2018; Agarwal et al., 2020).

Furthermore, they significantly increased plant fitness parameters such as shoot and root length, fresh biomass of shoot and root of pea plants in comparison to non-inoculated plants. A possible major mechanism behind the growth promoting effect by endophytic bacteria could be production of plant growth regulators such as indole acetic acid production and inorganic phosphate solubilization. The production of bacterial IAA modulates the intrinsic reserve of IAA in plants which in turn increase surface area of roots for better colonization, secretion of root exudates, and improvement of nutrient and water uptake efficiency (Shahzad et al., 2017; Gupta and Pandey, 2020). Similarly, the phosphate solubilization potential of biocontrol endophytic *Pseudomonas* and *Aneurinibacillus* strains proved to be beneficial for sustainable agriculture (Otieno et al., 2015; Chauhan et al., 2017).

The biocontrol of *F. oxysporum* root rot disease in pot trials indicated individual bacterial strains and consortium has significantly reduced the disease incidence in comparison to control. Similarly, endophytic bacteria from genera *Bacillus*, *Streptomyces* and *Burkholderia* suppressed the infectivity of *F. oxysporum* by 40 percent (Nwokolo et al., 2021).

The chlorophyll content was reduced drastically in control plants in comparison to endophytes primed plants under pathogen stress conditions. The pathogenic infection negatively influences photosynthetic process via decreasing electron transfer process during reaction, reducing chlorophyll content, minimizing the activity of carbon-fixation Ribulose-1,5-bisphosphate carboxylase-oxygenase (RuBisCo) enzyme as well as destructing thylakoids membrane proteins and lowering stomatal conductance during transpiration process in plants (Mishra et al., 2018). The chlorophyll content, however, in tested endophyte-pretreated pea plants was remarkably increased contrary to untreated pea plants in the presence of pathogen infection. The findings of the study are in accordance with the previous report of Mukherjee et al. (2020) which demonstrated enhanced chlorophyll content in chickpea plants inoculated with seed endophytes under *F. oxysporum* f.sp. *ciceris* induced biotic stress conditions. Similarly, the consortium treated pea plants showed increase carotenoid content suggesting the efficacy of endophytes in mitigation of toxic effects of pathogen inoculation on photosynthesis mechanism in plants as per Pandey et al. (2018).

Results suggested that prior presence of *P. aeruginosa* OS_12 and *A. aneurinilyticus* OS_25 confer tolerance to *Fusarium* root rot by meditating host resistance through defense related enzymes including PAL, polyphenol oxidase (PPO), PO), AO, CAT, and phenols. These pathogenesis related enzymes play a promising role in suppressing the pathogenic growth and development of associated symptoms in crop plants. Pre-treatment of plants with endophytes prompted the host plant to mediate rapid and more potent resistance upon encounter with phytopathogens (Sahu et al., 2019).

We observed that *Fusarium* challenged pea plants pre-treated either with individual endophytes OS_12 and OS_25 or consortia (OS_12 + OS_25) exhibited enhanced activity of PAL in contrary to uninoculated plants. Phenylalanine ammonia lyase (PAL; EC 4.3.1.24) is a major enzyme of the phenylpropanoid pathway responsible for synthesizing various defense related metabolites such as lignans, flavonoids, and isoflavonoids (Solekha et al., 2020). A parallel relationship has been observed between increased PAL activity and enhanced accumulation of phenolic compounds in pea plants infected with root rot pathogen. Phenolics accumulation is the key defense mechanism in plants in response to pathogen infection as reported in previous work by Singh and Gaur (2017) in chickpea crop. Similarly, peroxidase and polyphenol oxidase enzymatic activity were increased in fungal challenged pea plants under plant growth promoting endophyte treatment in agreement with previous studies by Muthukumar and Venkatesh (2014) and Khodadadi et al. (2016). Polyphenol oxidase (PPO; EC 1.10.3.1), a copper metalloenzyme oxidize phenolics to toxic and highly reactive o-quinones which either induce direct toxicity to pathogens or render low bioavailability of protein and create a physical barrier to pathogen attack (Zhang and Sun, 2021). Peroxidase (PO; EC 1.11.1.x) is another key enzyme of the defense response of plants to pathogen infection which contributes to scavenging pathogen induced reactive oxygen species (ROS), thus conferring tolerance to phytopathogen attack induced oxidative stress and protecting cellular components from oxidative burst. In a similar way, higher activity of ascorbate peroxidase was significantly higher in plants pre-treated with tested antagonistic bacterial strains under *Fusarium* induced biotic stress conditions with respect to other studied treatments. The findings are in accordance with previous reports which demonstrated the role of ascorbate peroxidase in ROS detoxification in plants to pathogen exposure (Agrawal et al., 2003). Catalase (CAT, EC 1.11.1.6) is another antioxidative key H_2_O_2_ detoxifying enzyme nullifies its detrimental effect on plant tissues and system on pathogen invasion (Yang and Poovaiah, 2002).

Additionally, the consortium treated plants had efficiently reduced ROS induced oxidative stress which was evident through lower amounts of MDA levels in comparison to infected control plants. This protective role might be due to PGPE mediated enhancement of ROS-scavenging antioxidant enzymatic activities, thus results in reduced lipid peroxidation under pathogen challenge (Ray et al., 2016).

The principal component analysis (PCA) was further carried out taking all the results of the present study into account which clearly showed the maximum aggregation of variables including morphological growth factors (length and fresh biomass of roots and shoots), antioxidants (CAT, TPC, and AO), defense related enzymes (PAL, PPO, and PO) as well as photosynthetic pigments (Chlorophyll and Carotenoids) content in consortium treated plants under both with and without pathogen challenged conditions.

The colonization of pea roots by *O. sanctum* endophytes- *P. aeruginosa* OS_12 and *A. aneurinilyticus* OS_25 was visualized using scanning electron microscopy and fluorescent microscopy. This association between bacteria and plants roots might be due to the root exudates secreted from plant roots, responsible for the chemotaxis and attachment of bacterial endophytes on to the root surface (rhizoplane) as well as subsequent proliferation of bacterial endophytes which ultimately leads to extensive colonization of roots (Liu et al., 2017; Saleh et al., 2020). Apart from these, most importantly, endophytic bacteria *P. aeruginosa* OS_12 and *A. aneurinilyticus* OS_25 isolated from leaves tissues of *O. sanctum* Linn. were able to colonize inner root section of *Pisum sativum* which implies the symbiotic characteristic of bacterial strains and thus has major biotechnological implications in sustainable agriculture.

The results of *in planta* experiments revealed that the consortium of *P. aeruginosa* OS_12 and *A. aneurinilyticus* OS_25 conferred significant enhancement of plant growth and suppression of disease pathogenesis in pea plants under both normal and *Fusarium* root rot pathogen challenged conditions in contrary to individual application. This increased PGP potential and biocontrol efficacy of consortium suggests that both the strains were compatible to each other and synergistically provide protection to pea plants against fungal root rot infection. Previous reports have demonstrated the effectiveness of consortia in conferring greater protection and PGP effects in various crop plants under biotic stressed conditions (Faria et al., 2021; Win et al., 2021).

## Conclusion

Results of present analysis suggest that endophytic bacteria *P. aeruginosa* OS_12 and *A. aneurinilyticus* OS_25 from healthy leaves of *O. sanctum* Linn. has played a significant role in improving plant health and performance under biotic stress. The mechanism employed by bacterial antagonists might be attributed to multiple factors such as direct phytopathogen inhibition through production of secondary metabolites including VOCs, siderophore, ammonia, and nutrient enrichment through solubilization of inorganic inaccessible fixed phosphate sources. Other indirect factors include modulation of innate antioxidants system triggering the production of defensive compounds inside the plant leading to suppression of pathogen growth and controlling its associated disease. Apart from these, most importantly, endophytic bacteria *P. aeruginosa* OS_12 and *A. aneurinilyticus* OS_25 isolated from the leaf tissues of *O. sanctum* Linn. were able to colonize inner root section of *P. sativum* which implies the symbiotic characteristic of bacterial strains and thus has major biotechnological implications in sustainable agriculture.

These bio-efficient endophytes could be a promising alternative to reduce the pathogenicity of root rot disease and improve the health of pea plants under *F. oxysporum* stress. However, for future prospects, further exploration is needed to elucidate the bio efficacy and plant growth promotion potential of these endophytes under actual field trials.

## Data Availability Statement

The datasets presented in this study can be found in online repositories. The names of the repository/repositories and accession number(s) can be found in the article/Supplementary Material.

## Author Contributions

SG conducted all the experiments under the guidance of SP. SP conceived and designed the research. SG and SP analyzed the data and wrote the manuscript. All authors contributed to the article and approved the submitted version.

## Conflict of Interest

The authors declare that the research was conducted in the absence of any commercial or financial relationships that could be construed as a potential conflict of interest.

## Publisher’s Note

All claims expressed in this article are solely those of the authors and do not necessarily represent those of their affiliated organizations, or those of the publisher, the editors and the reviewers. Any product that may be evaluated in this article, or claim that may be made by its manufacturer, is not guaranteed or endorsed by the publisher.

## Footnotes

1
http://www.ezbiocloud.net/eztaxon

2
https://www.ezbiocloud.net
