# Supplementary material for: Decoding the Plant Growth Promotion and Antagonistic Potential of Bacterial Endophytes From Ocimum sanctum Linn. Against Root Rot Pathogen Fusarium oxysporum in Pisum sativum
Source: Front Plant Sci. 2022 Feb 14;13:813686. doi: 10.3389/fpls.2022.813686 (PMC8884270; doi:10.3389/fpls.2022.813686)
Supplement: Supplementary file 1 [file Data_Sheet_1.docx]

**Decoding the plant growth promotion and antagonistic potential of bacterial endophytes from *Ocimum sanctum* Linn. against root rot pathogen *Fusarium oxysporum* in *Pisum sativum***

**Shikha Gupta^1^, Sangeeta Pandey^2^*, Satyawati Sharma^3^**

**^1^Amity Institute of Biotechnology, Amity University Uttar Pradesh, Sector 125, Noida, Uttar Pradesh 201313**

**^2^Amity Institute of Organic Agriculture, Amity University Uttar Pradesh, Sector 125, Noida, Uttar Pradesh 201313**

**^3^Centre for Rural Development and Technology, IIT Delhi, Hauz Khas, Delhi 110016, India**

**^*^Corresponding Author: Sangeeta Pandey; Email:** [**spandey5@amity.edu**](mailto:spandey5@amity.edu)**, sangeetamicro@gmail.com**

**Supplementary Table 1.** Volatile compounds detected by gas chromatography–mass spectrometry from endophytic bacterial strains OS_12 and OS_25

| **Tentative Compounds** | **Area (%)** | **RT (min)** | **Role** | **Reference** |
| --- | --- | --- | --- | --- |
| **Strain OS_12** | | | | |
| Dodecanoic acid (C_12_H_24_O_2_) | 2.78 | 10.68 | Antifungal activity | Walters et al., 2003; Al-Rashdi et al., 2020; Řiháková et al., 2001 |
| Tetra decanoic acid (C_14_H_28_O_2_) | 5.26 | 14.51 | Antifungal, Antimicrobial and indirectly promote salt stress tolerance | de Jonge et al., 2000; Agoramoorthy et al., 2007 |
| L-Ascorbic acid (C_6_H_8_O_6_) | 55.49 | 19.11 | Antioxidant, Plant growth promoting, provide resistance to pathogenic attack and suppresses pathogenesis | Boubakri, 2017 |
| Trans-13-Octadecanoic acid (C_18_H_34_O_2_) | 6.36 | 22.81 | Pharmacological activity:  anti-inflammatory activity | Hussein et al., 2017 |
| Octadecanoic acid (C_18_H_36_O_2_) | 30.10 | 23.42 | Antioxidant, Antimicrobial activity | Henry et al., 2002 |
| **Strain OS_25** | | | | |
| 2,2,3,4-Tetramethylpentane (C_9_H_20_) | 40.38 | 3.30 | - | - |
| (S)-3,4-Dimethylpentanol (C_7_H_16_O) | 23.72 | 3.77 | - | - |
| 1-aminoacetyl-Piperazine (C_6_H_13_N_3_O) | 24.29 | 3.92 | - | - |
| Octadecanoic acid (C_18_H_36_O_2_) | 4.49 | 27.68 | Antioxidant, Antimicrobial activity | Henry et al., 2002 |

**Supplementary Figure 1:** Map of National Capital Territory of Delhi, India with black dot indicating the soil sampling site

**Supplementary Figure 2:** Compatibility test between bacterial endophytes OS_12 and OS_25 was tested on nutrient agar media (a) by dual culture method (b) disk diffusion method (c) cross-streak method

**Supplementary Figure 3:** Effect of *Pseudomonas* *aeruginosa* OS_12 and *Aneurinibacillus* *aneurinilyticus* OS_25 inoculation on the germination of pea (*Pisum* *sativum*) after 10 days of sowing at 28 °C under normal conditions.

**Supplementary Figure 4:** Effect of *Pseudomonas* *aeruginosa* OS_12 and *Aneurinibacillus* *aneurinilyticus* OS_25 inoculation on the growth of pea plants sown in plastic pots with soil with (A) and without (B) *F. oxysporum* infection


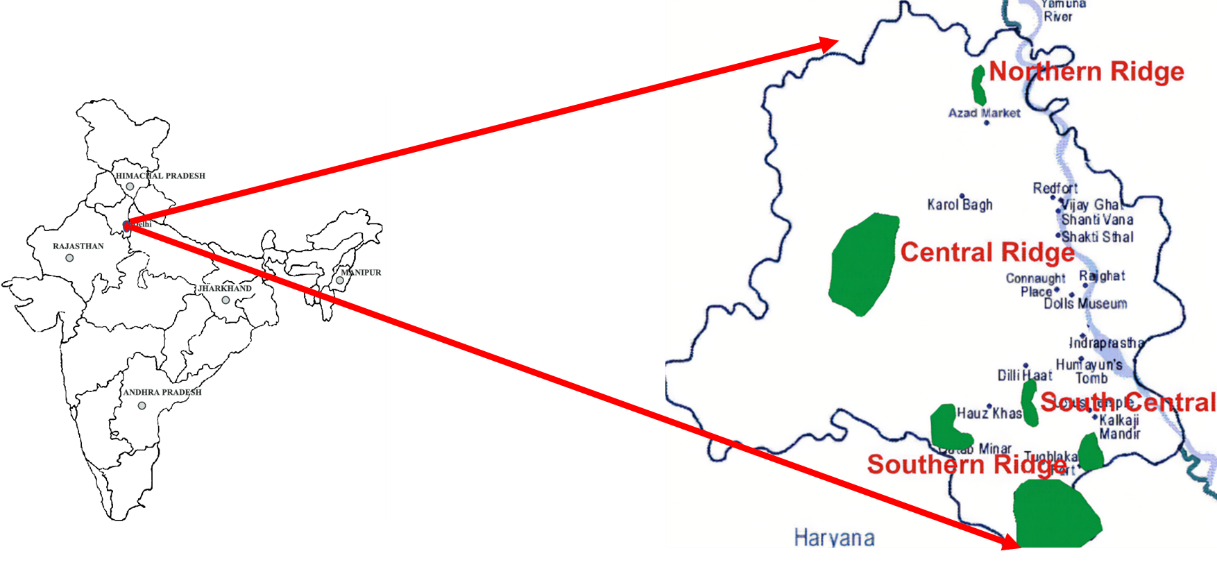


**Sanjay Van**

**Supplementary Figure 1:** Map of National Capital Territory of Delhi, India with black dot indicating the soil sampling site


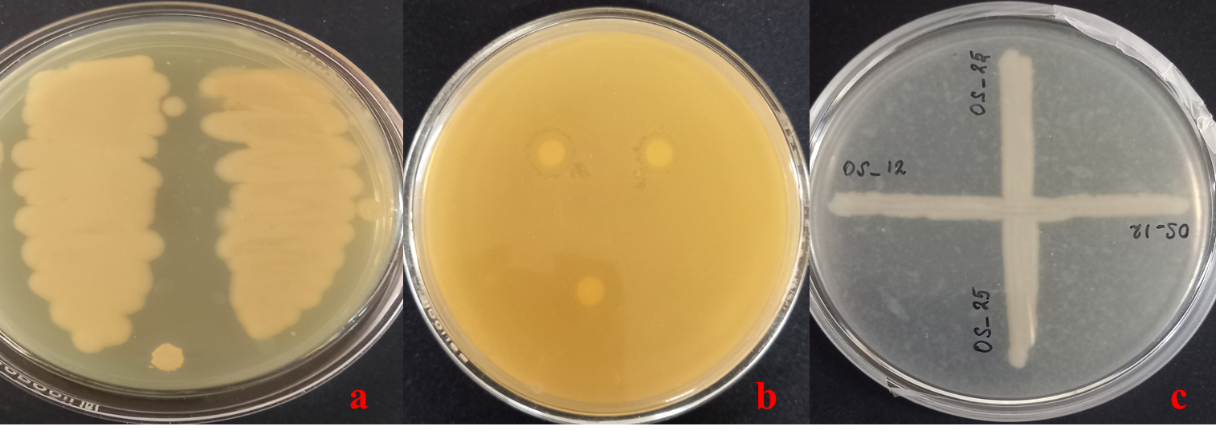


**Supplementary Figure 2:** Compatibility test between bacterial endophytes OS_12 and OS_25 was tested on nutrient agar media (a) by dual culture method (b) disk diffusion method (c) cross-streak method


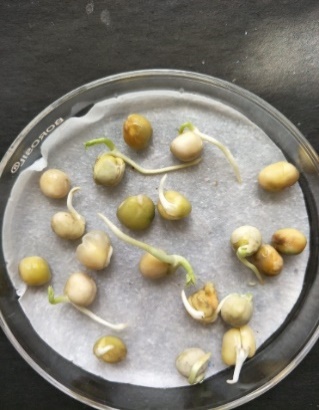

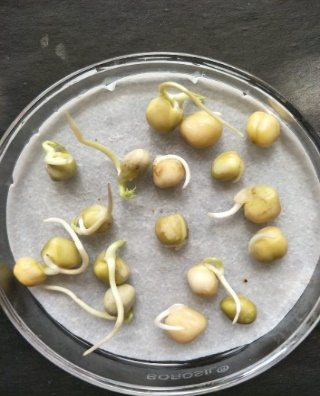

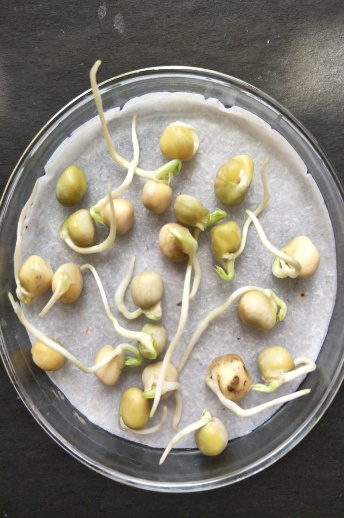

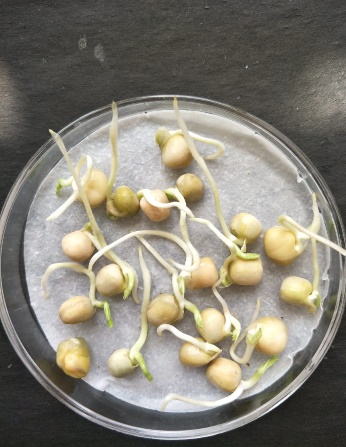

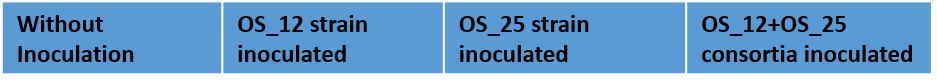


**Supplementary Figure 2:** Effect of *Pseudomonas* *aeruginosa* OS_12 and *Aneurinibacillus* *aneurinilyticus* OS_25 inoculation on the germination of pea (*Pisum* *sativum*) after 10 days of sowing at 28 °C under normal conditions.


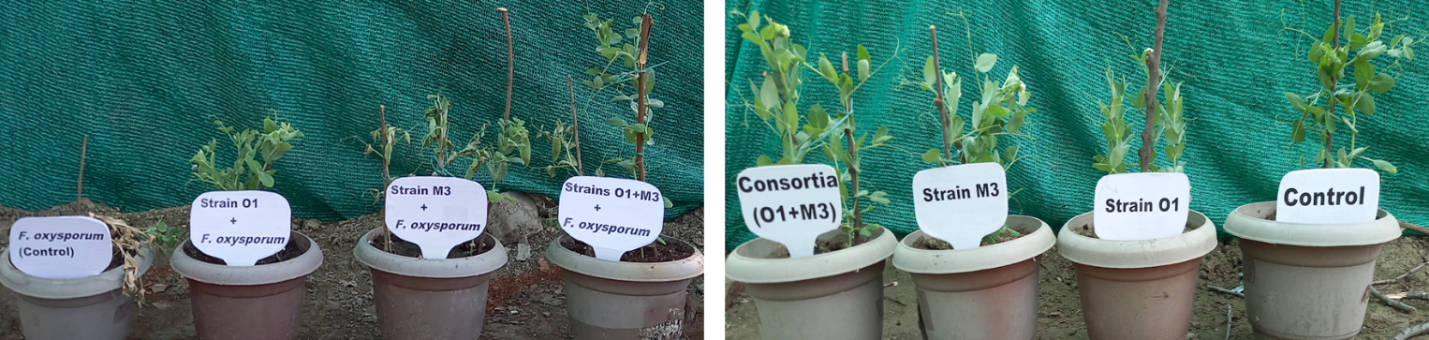


A.

B.

**Supplementary Figure 3:** Effect of *Pseudomonas* *aeruginosa* OS_12 and *Aneurinibacillus* *aneurinilyticus* OS_25 inoculation on the growth of pea plants sown in plastic pots with soil with (A) and without (B) *F. oxysporum* infection
